# Are children with early literacy difficulties at risk for anxiety disorders in late childhood?

**DOI:** 10.1007/s11881-023-00291-7

**Published:** 2023-11-08

**Authors:** Apostolos Kargiotidis, George Manolitsis

**Affiliations:** Present Address: Department of Preschool Education, University of Crete, 74100 Rethymno, Crete Greece

**Keywords:** Generalized anxiety, Literacy difficulties, Reading difficulties, Social anxiety, Spelling difficulties

## Abstract

The present study examined whether literacy difficulties in both grades 2 and 3 are associated with social and generalized anxiety within the school environment in grade 5 and if children with different literacy difficulties differ in anxiety levels compared to typically developing children in grade 5 after controlling for inattention. Sixty-nine Greek children with literacy difficulties and fifty-two children with typical literacy development were assessed at the beginning of grade 2 and at the end of grade 3 on standardized literacy measures (reading accuracy, text-reading fluency, reading comprehension, and spelling). In grade 5, teachers were asked to rate their children’s social and generalized anxiety levels and inattentive behavior in the school context. Results of one-way ANCOVAs showed that children with literacy difficulties were experiencing more social anxiety than typically developing children. Furthermore, children with both reading and spelling difficulties, but not those with single reading or spelling difficulties, had more social anxiety. These findings suggest that there is a close connection between early literacy difficulties and social anxiety in upper elementary grades and particularly among children with both reading and spelling difficulties. Implications for both teachers and other professionals who support children’s socioemotional development will be discussed.

A considerable proportion of primary school children faces significant challenges in learning to read and/or spell (Moll et al., 2014) with literacy difficulties (LD) being a risk factor not only for children’s academic success but also for their mental health (Livingston et al., 2018; McArthur et al., 2021). For instance, several studies have shown that children with LD are more likely to experience anxiety symptoms than typically developing (TD) children (Carroll et al., 2005; Mammarella et al., 2016; Novita et al., 2019; see also Georgiou & Parrila, 2023, for a review). These findings are also supported by a recent meta-analysis that showed a moderate association between children’s reading difficulties and anxiety (Francis et al., 2019). The anxiety symptoms of children with LD might be even more pronounced in the school context (Novita, 2016) because either they are afraid of school failure (Hendren et al., 2018) or they feel that they will be criticized by their classmates for their poor literacy skills when giving a presentation or reading aloud in class (Zuppardo et al., 2021). However, the exact mechanisms behind this association remain relatively unspecified (McArthur et al., 2021) because several factors may act as moderators (Francis et al., 2019).

One of these factors is the heterogeneity of both anxiety disorder and LD with researchers suggesting that specific LD subtypes may be related to specific types of anxiety symptoms (Francis et al., 2022; McArthur, 2022). However, research findings are quite inconsistent so far, and additional research is required to disentangle this complex relationship. For example, Grills-Taquechel et al. (2012) showed that it was fluency and not decoding difficulties that predicted higher separation anxiety. On the other hand, Francis et al. (2022) showed that among various reading skills and anxiety subtypes, there was only a negative correlation between reading accuracy and social anxiety. The meta-analysis by Francis et al. (2019) complicates things even more because they found that the type of reading difficulties did not moderate the association between reading difficulties and anxiety symptoms. Further research is also necessary because, to the best of our knowledge, the existing studies focus only on reading difficulties neglecting the potential role of children’s spelling difficulties. Research evidence shows that children with LD might present both reading and spelling difficulties (RSD), single-reading difficulties (RD), or single-spelling difficulties (SD) (Kargiotidis et al., 2021; Moll & Landerl, 2009) and that children with RSD experience the most severe cognitive deficits compared to TD children (Moll & Landerl, 2009). Therefore, it is important to examine whether a similar pattern would be observed regarding potential differences in anxiety levels and specific anxiety subtypes.

Finally, another variable that might moderate the association between children’s LD and anxiety symptoms is their attention level (McArthur et al., 2021). Studies have shown that attention deficits present significant comorbidity with anxiety disorder (e.g., Bowen et al., 2008; Costello et al., 2005; Tannock, 2008) and with LD (Kempe et al., 2011). Further, McArthur et al.’s (2021) results suggest that children with comorbid LD and anxiety are more likely to have poor attention.

The main aim of the present longitudinal study was to examine whether children with LD in both grades 2 and 3 will experience more social and generalized anxiety than TD children within the school environment in grade 5 after controlling for inattention. In addition, we examine whether children with different LD subtypes would differ in anxiety levels compared to TD children.

## Literacy difficulties and anxiety disorders

There is a growing concern among educational researchers, educators, and mental health workers about the adverse consequences of LD on children’s mental health (McArthur et al., 2021). One of the main reasons for this concern is the accumulated research evidence indicating that children with LD are at high risk for anxiety (Carroll et al., 2005; Francis et al., 2019; Mammarella et al., 2016; Zuppardo et al., 2021). However, children’s anxiety symptoms might vary (e.g., social anxiety, generalized anxiety, and separation anxiety) and research findings do not always coincide (McArthur et al., 2021).

For example, Mammarella et al. (2016) showed that 8- to 11-year-old children with reading difficulties experienced higher levels of social anxiety than TD children, whereas Grills-Taquechel et al. (2012) failed to find an association between reading difficulties and social anxiety with children in grade 1. The divergence between the two studies could be attributed to the age of the participants because the prevalence of social anxiety tends to increase as children get older (Costello et al., 2003; Lawrence et al., 2015). The core symptom of social anxiety is the fear of public exposure (American Psychiatric Association, 2013); therefore, it sounds reasonable for older children with LD to feel afraid to read aloud in front of their peers because they might think that they will be negatively criticized or even rejected (Zuppardo et al., 2021).

Recent studies have started to explore whether the association between LD and anxiety disorder is moderated by the type of anxiety symptoms or the type of LD (McArthur et al., 2021). Zuppardo et al. (2021) found that Italian grades 2–6 children with dyslexia presented higher levels of social and separation anxiety than TD children. What is more interesting is that social anxiety was found to be negatively associated with children’s performance on reading fluency and reading comprehension. According to Zuppardo et al. (2021), dyslexia is mainly manifested by slow reading fluency in highly transparent orthographies (like Italian and Greek) which then affects reading comprehension. Thus, children with LD might feel socially anxious when they have to perform reading activities within a time constraint. However, Grills-Taquechel et al. (2012) found that first-grade children who struggle with reading fluency are more likely to experience increased separation anxiety and not social anxiety. Their findings could be attributed to the participants’ age because the transition to primary school and the separation from their parents is considered to cause greater stress to first-grade children than their peers’ opinion about their reading ability.

Francis et al. (2022) assessed English-speaking children with and without reading difficulties from second to sixth grade οn a wide range of reading skills and anxiety symptoms. Their results revealed a significant, although weak, negative association between social anxiety and reading accuracy. No other significant association was found between the other reading skills (e.g., reading fluency and comprehension) and anxiety types (e.g., generalized and separation anxiety). Although the link between children’s LD and social anxiety seems to be corroborated again, the underlying mechanisms responsible for the association between social anxiety and reading accuracy, as opposed to reading fluency and reading comprehension, are not known.

Furthermore, Carroll et al. (2005) identified children with LD based on their performance on reading and spelling measures. To the best of our knowledge, this is the only study that takes into account both the reading and spelling skills of children with LD. Results showed that children with LD aged 9 to 15 experienced higher levels of generalized and separation anxiety but not social anxiety. The close association between LD and generalized and separation anxiety is not a novel finding because similar findings have been reported by other studies (e.g., Mammarella et al., 2016; Novita et al., 2019). However, Francis et al. (2022) have stated that the absence of a significant association between children’s LD and social anxiety in the study of Carroll et al. (2005) could be attributed to the low prevalence rates of social anxiety in their sample. Taking into consideration the well-established dissociation between children’s reading and spelling skills (Kargiotidis et al., 2021; Moll & Landerl, 2009), it would have been interesting if Carroll et al. (2005) had further classified their sample to children with RSD, children with RD, and children with SD to assess potential differences in anxiety symptoms compared to TD children.

Although the previous research findings depict a close link between LD and anxiety symptoms, they do not seem to agree on whether specific types of LD are associated with specific types of anxiety symptoms. The meta-analysis by Francis et al. (2019) did not support a possible association because they showed that the type of reading difficulties did not moderate the association between reading difficulties and anxiety symptoms. McArthur et al. (2021) have suggested that this inconsistency could be the outcome of methodological differences between studies, including the transparency of the orthography, the age of the participants, and the selection criteria of at-risk populations. Therefore, further research is needed to provide additional information.

## The role of inattention in the association between LD and anxiety disorders

It is well-established that LD are often accompanied by poor attentional levels, possibly because several of their cognitive predictors share common underlying genetic factors (Hendren et al., 2018). Research evidence has shown that the attention difficulties of children with LD are specific rather than pervasive (Lewandowska et al., 2014), and they might be present even before the beginning of formal reading instruction (Parhiala et al., 2015). For instance, children with LD are more likely to struggle to focus and keep their attention on a particular school task, and they are expected to get more easily distracted (Marzocchi et al., 2009). Also, their attention is less flexible and that might make it hard for them to shift their focus from one word to another when they are reading a passage (Lewandowska et al., 2014).

According to the *Attentional Control Theory*, anxiety interferes with attentional resources which are necessary for learning (Eysenck et al., 2007). For example, children with anxiety symptoms focus their attention to stressful thoughts (e.g., what my classmates will think of me if they hear me reading?) and cannot concentrate to successfully accomplish a learning task. As a result, they are not able to fully attend and participate in the learning process, and their school performance is negatively affected (Massetti et al., 2008). Similarly, in McArthur’s (2022) *Poor Reading and Anxiety* (PRAX) model, inattention is described as a “maintenance variable” that leads from anxiety to reading difficulties. Grills-Taquechel et al. (2013) showed that those children who feel more anxious to perform well on school assignments and are less attentive are more likely to present reading fluency difficulties. On the other hand, when inattentiveness was combined with low separation anxiety levels, children performed worse on reading fluency. However, a considerable number of studies advocate in favor of a significant association between LD and anxiety symptoms, even after accounting for the effects of inattention (Arnold et al., 2005; Carroll et al., 2005; Francis et al., 2022; Hossain et al., 2021). Furthermore, in the meta-analysis by Francis et al. (2019), although reading difficulties, anxiety, and inattention were associated with each other, inattention did not seem to play a moderating role. Therefore, to better understand the association between LD and anxiety symptoms, it is important to examine the role of inattention as a covariate and whether significant associations remain between different anxiety and LD subtypes.

## The present study

The present study is part of a broader longitudinal project and aims to examine whether children with LD in both grades 2 and 3 will experience more social and generalized anxiety in grade 5 than TD children after controlling for inattention. Moreover, we examined whether children with different LD subtypes (RSD, RD, and SD) will differ in anxiety levels compared to TD children in grade 5 after controlling for inattention. The current study aimed to answer the following research questions:

1. Do children with LD in both grades 2 and 3 experience increased levels of social and generalized anxiety compared to TD children after controlling for inattention?

Based on previous research findings (Mammarella et al., 2016; Novita et al., 2019; Zuppardo et al., 2021), we hypothesized that children with LD would experience more social and generalized anxiety than TD children in grade 5.

(2) Do children with different LD subtypes (RSD, RD, and SD) in both grades 2 and 3 experience more anxiety symptoms than TD children in grade 5 after controlling for inattention?

Although a few studies have indicated that children with specific reading difficulties subtypes might experience more anxiety symptoms than TD children (e.g., Francis et al., 2022; Grills-Taquechel et al., 2012), no study, as far as we know, has assessed whether children with either both reading and spelling difficulties (RSD) or single reading (RD) or spelling (SD) difficulties experience higher anxiety levels than TD children. However, it has been shown that among children with different LD subtypes, children with RSD present the most deficient cognitive profile compared to TD children (Moll & Landerl, 2009). Thus, we hypothesized that this might cause them greater stress, and as a result, their teachers would rate them as experiencing more anxiety symptoms than TD children.

## Method

### Participants

The participants of the present longitudinal study were 121 children (53 girls) who attended public mainstream elementary schools in Heraklion, Greece, from grade 2 to grade 5. Participants took part in a broader longitudinal project and they were initially selected when they were in grade 1 (see Kargiotidis et al., 2021 for more details regarding participant selection process). All children were Greek native speakers with written parental consent, they had no formal diagnosis of intellectual, neurodevelopmental, or sensory disorder, and they had typical non-verbal ability (i.e., Raven’s score above 70).

### Measures

#### Non-verbal intelligence

The Greek standardization of the Raven’s Colored Progressive Matrices was used to assess non-verbal intelligence (Raven, 1956; Sideridis et al., 2015). Children were asked to choose among six alternatives the one that best completed a presented matrix with a part missing. Cronbach’s alpha in the standardization sample was 0.90 (Sideridis et al., 2015).

#### Word reading accuracy

The *Word Decoding* and the *Pseudoword Decoding* subscales of a Greek standardized reading scale were administered to assess word reading accuracy (Padeliadu et al., 2019). In the Word Decoding subscale, a page with 57 words arranged in increasing order of difficulty was presented to children, and they were instructed to read them with no time limit. Similarly, in the Pseudoword Decoding subscale, children were asked to read a series of 40 pseudowords arranged in ascending order of difficulty without any time constraint. In both subscales, testing was terminated if a child made five consecutive errors. Cronbach’s alpha in our sample was for the Word Decoding subscale 0.92 in grade 2 and 0.88 in grade 3, and for the Pseudoword Decoding subscale 0.86 in grade 2 and 0.86 in grade 3.

#### Reading fluency

The *Text-Reading Fluency* subscale of a Greek standardized reading measure (Padeliadu et al., 2019) was used for the assessment of reading fluency. Participants were asked to read a passage of 247 words that referred to an ancient Greek myth as fast and accurately as possible in 1 min. The total score for each participant was the number of words that were read correctly within 1 min. Test–retest reliability in the standardization study was 0.98 (Padeliadu et al., 2019).

#### Reading comprehension

The Greek standardized “Screening Test of Reading Ability” (Tafa, 1995) was administered to measure reading comprehension. It includes 42 sentences each of which is missing one word. Children had to read each sentence and select among four alternative words the one that correctly completed the sentence in a time of 40 min. Cronbach’s alpha in our sample was 0.77 in grade 2 and 0.89 in grade 3.

#### Spelling

A standardized spelling test (Mouzaki et al., 2007) was used for the assessment of children’s spelling skills. Children were asked to write 60 words arranged in ascending order of difficulty which were presented orally to them in three steps. Particularly, each word was initially read aloud in isolation, then as a part of a sentence, and then again in isolation. After hearing the word in isolation for a second time, children were instructed to write it down. If a child made six consecutive misspellings, testing was terminated, and his/her score was the total number of correctly spelled words. Cronbach’s alpha in our sample was 0.83 in grade 2 and 0.93 in grade 3.

#### Anxiety

To assess children’s anxiety in the school environment, we adapted in Greek the School Anxiety Scale-Teacher Report (SAS-TR; Lyneham et al., 2008) which comprises 16 items divided into two subscales: 7 items for social anxiety (e.g., “This child is afraid of asking questions in class,” “This child speaks only when someone asks a question of them”) and 9 items for generalized anxiety (e.g., “This child is afraid of making mistakes,” “This child worries about things”). Classroom teachers rated on a 4-point Likert scale ranging from 0 to 3 (0 = “Never” and 3 = “Always”) how the child’s behavior had been this school year. The potential maximum score for the social anxiety subscale was 21 and for the generalized anxiety subscale 27. For the subsequent analyses, the average scores for children’s social and generalized anxiety were created. Cronbach’s alpha of the social anxiety and the generalized anxiety subscale in our sample was 0.85 and 0.77, respectively.

#### Inattention

To assess children’s inattention, we adapted in Greek the inattention subscale of the fourth edition of the Swanson, Nolan, and Pelham ADHD rating scale (SNAP-IV; Swanson et al., 2012). The SNAP-IV is an 18-item parent/teacher-report scale that assesses children’s inattention (9 items) and hyperactivity-impulsivity (9 items). For the purpose of the present study, we used only the nine items of the inattention subscale. The items were completed by children’s classroom teachers who were asked to circle on a 4-point Likert scale ranging from 0 to 3 (0 = “Not at all” and 3 = “Very Much”) the option that best described the child’s behavior during the current school year. The potential maximum score for the inattention subscale was 27 and in the analyses that followed the average score of inattention was used. Cronbach’s alpha of the inattention subscale in the current sample was 0.96.

### Procedure

Children in the present study were assessed by (a) trained research assistants (postgraduate students of psychology or education) in grades 2 and 3 and (b) their classroom teachers in grade 5. Specifically, in the middle of grade 1, children’s non-verbal intelligence was measured together with other cognitive skills (for the needs of the broader longitudinal project) in a 20-min individual session. At the beginning of grade 2 and at the end of grade 3, children’s reading and spelling skills were assessed in one individual and one group session. In the individual sessions, we assessed children’s word reading accuracy, reading fluency, and spelling. In the group-sessions, we measured reading comprehension in groups of approximately 10 children each. Finally, in grade 5, we administered to children’s classroom teachers a teacher-report questionnaire to rate children’s anxiety and inattention levels. All activities were conducted in compliance with the ethical guidelines of the American Psychological Association and the Declaration of Helsinki for research involving human subjects. The study was also approved by the Research Ethics Committee of the Department of Preschool Education of the University of Crete.

### Statistical analysis

Initially, children were classified into LD and TD group based on their performance on standardized measures of word reading accuracy, reading fluency, reading comprehension, and spelling at the beginning of grade 2 and at the end of grade 3. The LD group (*N* = 69; 32 girls) consisted of those children who scored below the 25th percentile on at least one reading test and/or the spelling test in both grades. The remaining children were assigned to the TD group (*N* = 52; 21 girls). Children with LD performed significantly lower than TD children on all literacy measures in grades 2 and 3 (see Table 1).

**Table 1:** Means (M) and standard deviations (SD) for all literacy measures assessed in grades 2 and 3 for the LD and the TD group

Measures	LD group				TD group				*t* test^1^ Grade 2	*t* test^1^ Grade 3
	Grade 2		Grade 3		Grade 2		Grade 3			
	*M*	SD	*M*	SD	*M*	SD	*M*	SD		
Word decoding	33.75	10.75	42.42	7.38	44.63	4.96	49.60	4.24	7.43** *d* = 1.30	6.73** *d* = 1.19
Pseudoword decoding	22.77	5.95	24.16	4.58	27.12	5.35	31.02	4.60	4.15** *d* = .76	8.14** *d* = 1.50
Text-reading fluency	35.75	12.78	70.96	20.82	56.52	15.73	104.96	20.15	8.01** *d* = 1.47	9.02** *d* = 1.67
Reading comprehension	13.59	4.80	23.13	7.26	18.88	6.18	31.12	6.48	5.12** *d* = .96	6.27** *d* = 1.15
Spelling	12.72	3.21	20.12	5.53	17.77	5.39	33.00	7.75	5.99** *d* = 1.14	10.67** *d* = 1.96

*LD* children with literacy difficulties, *TD* typically developing children

^**^p < .001

^1^Bonferroni correction was performed for 5 comparisons in both grades (*p* < .01)

We further classified children in the LD group into three subgroups. The first subgroup included children with both reading and spelling difficulties (RSD; *N* = 35; 21 girls) who scored below the 25th percentile on at least one reading test and on the spelling test in both grades. The second subgroup consisted of children with single spelling difficulties (SD; *N* = 18; 8 girls) who performed on the spelling test below the 25th percentile in both grades 2 and 3 and performed above the 25th percentile on reading tests in at least one of the two grades. Finally, the third subgroup included children with single-reading difficulties (RD; *N* = 16; 3 girls) who scored below the 25th percentile on at least one reading test in both grades 2 and 3 and performed above the 25th percentile on the spelling test in at least one of the two grades. Significant differences on all literacy measures in grades 2 and 3 among the three LD subgroups and between each of these groups and TD children are presented in Table 3.

Regarding the statistical analyses of the present study, first, we conducted a series of independent sample *t* tests and one-way analyses of variance (ANOVAs) to examine whether the various literacy groups (i.e., LD vs TD and RSD vs RD vs SD vs TD) differed from each other on literacy skills and on the levels of the anxiety symptoms and inattention. Second, to ensure that the potential differences on anxiety symptoms are associated with children’s literacy group classification and not to their levels of inattention, we run a series of one-way analyses of covariance (ANCOVAs) with anxiety symptoms (i.e., social anxiety and generalized anxiety) as the dependent variable and inattention as a covariate. Variable examination revealed no missing values or extreme outliers. Moreover, we conducted a series of correlational analyses calculating Pearson’s *r* coefficient to measure the interrelations between children’s anxiety symptoms and inattention.

## Results

### Preliminary analyses

Descriptive statistics for each literacy group for literacy skills in grades 2 and 3 and for anxiety symptoms and inattention in grade 5 are presented in Tables 1, 2, 3, and 4. Independent sample *t* tests with Bonferroni adjustment revealed that children with LD presented significantly higher levels of inattention and social anxiety than TD children in grade 5 but not of generalized anxiety (see Table 2).

**Table 2:** Means (M) and standard deviations (SD) for anxiety symptoms and inattention in grade 5 for the LD and the TD group

Measures	LD group		TD group		*t* test^1^
	*M*	SD	*M*	SD	
Social anxiety	0.83	0.58	0.49	0.47	3.50** *d* = .64
Generalized anxiety	0.54	0.35	0.41	0.33	2.09 *d* = .38
Inattention	1.02	0.72	0.55	0.70	3.56** *d* = .65

*LD* Children with literacy difficulties, *TD* typically developing children

^**^p < .001

^1^Bonferroni correction was performed for 3 comparisons (*p* < .017)

**Table 3:** Means (M) and standard deviations (SD) for all literacy measures assessed in grades 2 and 3 for the LD subgroups and the TD group

Measures	RSD group		RD group		SD group		TD group		ANOVA *F*-test df = 3, 117
	*M*	SD	*M*	SD	*M*	SD	*M*	SD	
Word decoding									
Grade 2	28.40^1^	11.14	39.31^2^	8.51	39.22^2^	5.52	44.63^2^	4.96	30.57***, η^2^_ρ_ = .44
Grade 3	37.69^1^	7.36	47.44^2^	3.22	47.17^2^	2.60	49.60^2^	4.24	40.12***, η^2^_ρ_ = .51
Pseudoword decoding									
Grade 2	20.74^1^	6.70	23.69^1,2^	4.83	25.89^2^	3.45	27.12^2^	5.35	9.84***, η^2^_ρ_ = .20
Grade 3	22.34^1^	3.95	25.50^1,3^	4.99	26.50^3^	4.06	31.02^2^	4.60	28.23***, η^2^_ρ_ = .42
Text-reading fluency									
Grade 2	29.17^1^	12.58	41.31^3^	9.95	43.61^3^	8.15	56.52^2^	15.73	30.11***, η^2^_ρ_ = .44
Grade 3	62.49^1^	19.74	79.06^3^	16.64	80.22^3^	20.22	104.96^2^	20.15	34.10***, η^2^_ρ_ = .47
Reading comprehension									
Grade 2	11.97^1^	4.70	13.88^1,2^	3.90	16.50^2,3^	4.50	18.88^3^	6.18	12.82***, η^2^_ρ_ = .25
Grade 3	19.86^1^	5.38	23.75^1,2^	6.68	28.94^2,3^	7.43	31.12^3^	6.48	23.73***, η^2^_ρ_ = .38
Spelling									
Grade 2	11.06^1^	2.70	15.69^2,3^	3.14	13.33^1,2^	1.91	17.77^3^	5.39	20.08***, η^2^_ρ_ = .34
Grade 3	17.89^1^	4.58	25.44^3^	5.25	19.72^1,3^	4.36	33.00^2^	7.75	48.00***, η^2^_ρ_ = .55

*RSD* children with reading and spelling difficulties, *RD* children with single reading difficulties, *SD* children with single spelling difficulties, *TD* typically developing children

^***^p < .001

^1^^,2,3^Superscript numbers refer to pairwise comparisons (Bonferroni). Means with at least one same superscript number do not differ significantly

**Table 4:** Means (M) and standard deviations (SD) for anxiety symptoms and inattention in grade 5 for the LD subgroups and the TD group

Measures	RSD group		RD group		SD group		TD group		ANOVA *F*-test df = 3, 117
	*M*	SD	*M*	SD	*M*	SD	*M*	SD	
Social anxiety	1.06^1^	0.57	0.59^2^	0.58	0.60^2^	0.43	0.49^2^	0.47	9.42***, η^2^_ρ_ = .19
Generalized anxiety	0.64^1^	0.34	0.40^1,2^	0.32	0.47^1,2^	0.33	0.41^2^	0.33	3.74*, η^2^_ρ_ = .09
Inattention	1.28^1^	0.63	0.79^1,2^	0.58	0.70^2^	0.82	0.55^2^	0.70	8.17***, η^2^_ρ_ = .17

*RSD* children with reading and spelling difficulties, *RD* children with single reading difficulties, *SD* children with single spelling difficulties, *TD* typically developing children

^*^p < .05

^***^p < .001

^1^^,2,^Superscript numbers refer to pairwise comparisons (Bonferroni). Means with at least one same superscript number do not differ significantly

A series of one-way ANOVAs showed statistically significant differences with small effect sizes among the three LD subgroups and between each of these groups and TD children on all anxiety and inattention measures in grade 5 (see Table 4). Post-hoc Bonferroni comparisons revealed that children with RSD were rated by their teachers as presenting higher levels of inattention than children with SD, *t* (117) = 2.94, *p* < 0.05, *d* = 0.84, and TD children, *t* (117) = 4.88, *p* < 0.001, *d* = 1.09. Furthermore, teachers reported that children with RSD had higher levels of social anxiety, *t* (117) = 3.16, *p* < 0.05, *d* = 0.89, than children with SD, children with RD, *t* (117) = 3.08, *p* < 0.05, *d* = 0.83, and TD children, *t* (117) = 5.17, *p* < 0.001, *d* = 1.13. Children with RSD had also higher levels of generalized anxiety, *t* (117) = 3.15, *p* < 0.05, *d* = 0.69, than TD children.

Finally, correlational analyses showed that both social and generalized anxiety were significantly correlated with inattention with moderate *r* coefficients (*r* = 0.43 for social anxiety–inattention and *r* = 0.49 for generalized anxiety–inattention).

### The association between LD and anxiety symptoms controlling for inattention

A one-way ANCOVA was conducted to assess whether the statistically significant difference that was found between children with LD and TD children on social anxiety would be maintained after controlling for inattention. Results revealed that there was still a significant main effect of literacy group on social anxiety, *F* (1, 118) = 5.02, *p* < 0.05, η^2^_ρ_ = 0.04, with children with LD being rated by their teachers as presenting higher levels of social anxiety than TD children.

Similarly, a series of one-way ANCOVAs was conducted to examine whether the statistically significant differences that were found among children with different LD subtypes and between them and TD children on anxiety symptoms would remain after controlling for inattention. Results showed that there was still a significant main effect of LD subgroup on social anxiety, *F* (3, 116) = 4.48, *p* < 0.01, η^2^_ρ_ = 0.10, but not on generalized anxiety, *F* (3, 116) = 0.77, *p* = 0.51, η^2^_ρ_ = 0.02. Post-hoc Bonferroni comparisons indicated that children with RSD were rated by their teachers’ as having higher levels of social anxiety, *t* (116) = 3.54, *p* < 0.01, *d* = 0.81, than children with TD. Any other significant between-group differences that there were found in the one-way ANOVAs were no longer present after controlling for inattention.

## Discussion

The present longitudinal study examined whether children with LD in both grades 2 and 3 would experience higher anxiety levels than TD children in grade 5 as well as if the teacher-reported anxiety symptoms would differ between children with different LD subtypes (RSD, RD, and SD) and TD children in grade 5 after controlling for inattention. Overall, our results showed that (a) children with LD were rated by their teachers as experiencing more social anxiety than TD children in grade 5 and (b) children with RSD were experiencing higher levels of social anxiety than TD children in grade 5 beyond the effects of inattention. Our findings are discussed below based on the formulated research questions and the corresponding research hypotheses.

### Differences in anxiety symptoms between children with LD and TD children

The current research findings provided partial support to our first hypothesis. Particularly, although it was expected that children with LD would be rated by their teachers as presenting higher levels of generalized and social anxiety than TD children after controlling for inattention, our results revealed that the only significant difference between the two groups was in social anxiety. A close association between LD and social anxiety has also been reported by Zuppardo et al. (2021) who found that children with dyslexia attending second to sixth grade are more likely to feel stressed when they are asked to read aloud in class because of their fear of being humiliated or rejected by their peers. Similarly, the results of Mammarella et al. (2016) have indicated that children with reading difficulties attending third to sixth grade experience more social anxiety than TD children. However, it should be mentioned that both of these studies employed a cross-sectional methodological design without controlling for the effects of inattention. Therefore, the present longitudinal findings extend the previous ones and provide valuable insights regarding the negative consequences of early LD on children’s mental health in late childhood.

Interestingly, our findings showed that, after controlling for inattention, children with early LD might feel particularly anxious in upper elementary grades when they have to participate in group activities or to ask/answer a question in front of their classmates. Children with LD might fear that they will be negatively evaluated or even rejected by their peers due to their poor academic performance on literacy tasks (Zuppardo et al., 2021). Empirical research has shown that children’s fears about social evaluation tend to increase with age (Westenberg et al., 2004), which in turn rises the frequency of socially anxious behavior after middle childhood (Poole et al., 2022). Therefore, children with LD might take into account their classmates’ judgments in late childhood, which might explain why LD were significantly associated with social anxiety and not with generalized anxiety in our study.

Furthermore, our results partially confirmed our second hypothesis. Particularly, they revealed that among children with different LD subtypes, children with RSD were experiencing higher social anxiety than TD children, which was not entirely accounted for by inattention. Previous studies have laid the ground, but our study is the first one, to the best of our knowledge, that examined the potential differences in specific anxiety symptoms between children with dissociated profiles of reading and spelling deficits and TD children. Our novel findings suggest that children with more severe LD, manifested by deficits in both reading and spelling, are at a great risk of social anxiety. Empirical research has shown that children’s RSD are associated with multiple cognitive deficits (Moll & Landerl, 2009), and they may also coexist with difficulties in other academic domains, such as mathematics (Slot et al., 2016). Therefore, it could be argued that because of the more pervasive difficulties that children with RSD face, they may feel particularly anxious when they have to engage in school activities that require social interaction or personal exposure.

Based on our second hypothesis, it was also expected that children with RSD would experience more generalized anxiety than TD children after controlling for inattention. Although our initial results showed that children with RSD had significantly higher levels of generalized anxiety than TD children in grade 5, when inattention was entered into the equation as a covariate, the significant effect disappeared. Based on the meta-analysis of Willcutt et al. (2012), which showed that inattention is more strongly correlated with generalized anxiety compared to other anxiety subtypes, and the suggestions of McArthur’s (2022) PRAX model, one possible explanation of our results could be that inattention might act as a mediating factor that connects children’s generalized anxiety to their poor literacy skills. However, additional research is needed to shed more light on this complex association.

## Limitations

The present study has some limitations. First, children’s anxiety symptoms were assessed only in grade 5; therefore, it was not possible to examine the direction of the association between anxiety and LD. It would be of particular interest if future studies assess children’s anxiety levels at the beginning of first grade to clarify whether children’s early LD trigger later anxiety symptoms or if children’s pre-existing anxiety symptoms cause their LD. Second, although we focused on two different types of anxiety symptoms, we did not examine reading anxiety, which is a promising but understudied area (Hendren et al., 2018). Third, our LD subtype groups were relatively small, and a future study should replicate our findings with a larger sample. Finally, another limitation of our study is that we relied solely on a teacher-report measure to assess children’s anxiety levels. Future research could also include self-report and parent-report anxiety measures.

## Educational implications

Our findings have significant psychoeducational implications for both teachers and mental health professionals who support children with LD. In line with previous research (Livingston et al., 2018), our study underlines once more that children with early LD are at increased risk of experiencing anxiety disorders in late childhood. Based on our results, their symptoms in upper elementary grades are mainly focused on social anxiety, possibly because of the increasing importance of social evaluations at this developmental stage (Westenberg et al., 2004). Therefore, interventions should focus not only on the improvement of these children’s literacy skills but also on the promotion of their self-esteem levels (Novita, 2016; Zuppardo et al., 2021) in order to protect their later mental health. Finally, our results suggest that children with RSD are in need of more intensive and broader interventions because apart from their pervasive cognitive and academic difficulties (Moll & Landerl, 2009; Slot et al., 2016), they are also more likely to present social anxiety. One promising treatment that seems to have multiple benefits is mindfulness meditation (Hendren et al., 2018). Research evidence has shown that it might improve literacy skills and decrease anxiety levels in children (Keller et al., 2019) and adolescents with LD (Beauchemin et al., 2008), while in the study by Tarrasch et al. (2016) with adults with dyslexia results revealed that it might also improve attention. However, more empirical evidence is needed about its effectiveness in children with LD.

## Conclusion

In summary, our findings highlighted again the adverse consequences that early LD might have on children’s later mental health. Children with LD in both grades 2 and 3 were experiencing more social anxiety than TD children in grade 5, which was not accounted for inattention. Notably, they seem to feel particularly stressed in situations where they have to talk in front of their classmates, and they hesitate to pose or answer a question in class. Our results revealed for the first time that children with RSD were experiencing more social anxiety than TD children in grade 5, even after controlling for inattention. Overall, in line with previous research evidence, our study corroborates the close connection between LD and anxiety beyond the effects of other significant correlates of LD, such as inattention, and enriches the existing literature with novel findings suggesting that specific LD subtypes might be associated with specific anxiety behaviors.

## References

[CR1] American Psychiatric Association (2013). Diagnostic and statistical manual of mental disorders.

[CR2] Arnold EM, Goldston DB, Walsh AD, Reboussin BA, Daniel SS, Hickman E, Wood F (2005). Severity of emotional and behavioral problems among poor and typical readers. Journal of Abnormal Child Psychology.

[CR3] Beauchemin J, Hutchins TL, Patterson F (2008). Mindfulness meditation may lessen anxiety, promote social skills, and improve academic performance among adolescents with learning disabilities. Complementary Health Practice Review.

[CR4] Bowen R, Chavira DA, Bailey KM, Stein MT, Stein MB (2008). Nature of anxiety comorbid with attention deficit hyperactivity disorder in children from a pediatric primary care setting. Psychiatry Research-Neuroimaging.

[CR5] Carroll JM, Maughan B, Goodman R, Meltzer H (2005). Literacy difficulties and psychiatric disorders: Evidence for comorbidity. Journal of Child Psychology and Psychiatry.

[CR6] Costello EJ, Mustillo SA, Erkanli A, Keeler G, Angold A (2003). Prevalence and development of psychiatric disorders in childhood and adolescence. Archives of General Psychiatry.

[CR7] Costello EJ, Egger HL, Angold A (2005). The developmental epidemiology of anxiety disorders: Phenomenology, prevalence, and comorbidity. Child and Adolescent Psychiatric Clinics of North America.

[CR8] Eysenck MW, Derakshan N, Santos R, Calvo MG (2007). Anxiety and cognitive performance: Attentional control theory. Emotion.

[CR9] Francis D, Caruana N, Hudson JL, McArthur G (2019). The association between poor reading and internalising problems: A systematic review and meta-analysis. Clinical Psychology Review.

[CR10] Francis DA, Hudson JL, Robidoux S, McArthur GM (2022). Are different reading problems associated with different anxiety types?. Applied Cognitive Psychology.

[CR11] Georgiou G, Parrila R, Friedman H, Markey C (2023). Dyslexia and mental health problems. Encyclopedia of mental health.

[CR12] Grills-Taquechel AE, Fletcher JM, Vaughn S, Stuebing KK (2012). Anxiety and reading difficulties in early elementary school: Evidence for unidirectional- or bi-directional relations?. Child Psychiatry & Human Development.

[CR13] Grills-Taquechel AE, Fletcher JM, Vaughn S, Denton CA, Taylor P (2013). Anxiety and inattention as predictors of achievement in early elementary school children. Anxiety Stress and Coping.

[CR14] Hendren, R. L., Haft, S. L., Black, J. M., White, N. J., & Hoeft, F. (2018). Recognizing psychiatric comorbidity with reading disorders. *Frontiers in Psychiatry*, *9*. 10.3389/fpsyt.2018.00101 10.3389/fpsyt.2018.00101 PMC5880915 29636707

[CR15] Hossain B, Bent S, Hendren RL (2021). The association between anxiety and academic performance in children with reading disorder: A longitudinal cohort study. Dyslexia.

[CR16] Kargiotidis A, Grigorakis I, Mouzaki A, Manolitsis G (2021). Differences in oral language growth between children with and without literacy difficulties: Evidence from early phases of learning to read and spell in Greek. Australian Journal of Learning Difficulties.

[CR17] Keller JH, Ruthruff E, Keller P (2019). Mindfulness and speed testing for children with learning disabilities: Oil and water?. Reading & Writing Quarterly.

[CR18] Kempe C, Gustafson S, Samuelsson S (2011). A longitudinal study of early reading difficulties and subsequent problem behaviors. Scandinavian Journal of Psychology.

[CR19] Lawrence, D., Johnson, S., Hafekost, J., Boterhoven de Haan, K., Sawyer, M., Ainley, J., & Zubrick, S. R. (2015). *The** mental health of children and adolescents: Report on the second Australian child and adolescent survey of mental health and wellbeing.* Commonwealth of Australia. Retrieved January 17, 2023, from https://www.health.gov.au/resources/publications/the-mental-health-of-children-and-adolescents

[CR20] Lewandowska, M., Milner, R., Ganc, M., Włodarczyk, E., & Skarżyński, H. (2014). Attention dysfunction subtypes of developmental dyslexia. *Medical Science Monitor*, *20*, 2256–2268. 10.12659/msm.890969 10.12659/MSM.890969 PMC4238793 25387479

[CR21] Livingston EM, Siegel LS, Ribary U (2018). Developmental dyslexia: Emotional impact and consequences. Australian Journal of Learning Difficulties.

[CR22] Lyneham HJ, Street A, Abbott MJ, Rapee RM (2008). Psychometric properties of the school anxiety scale—Teacher report (SAS-TR). Journal of Anxiety Disorders.

[CR23] Mammarella IC, Ghisi M, Bomba M, Bottesi G, Caviola S, Broggi F, Nacinovich R (2016). Anxiety and depression in children with nonverbal learning disabilities, reading disabilities, or typical development. Journal of Learning Disabilities.

[CR24] Marzocchi GL, Ornaghi S, Barboglio S (2009). What are the causes of the attention deficits observed in children with dyslexia?. Child Neuropsychology.

[CR25] Massetti GM, Lahey BB, Pelham WE, Loney J, Ehrhardt A, Lee SS, Kipp H (2008). Academic achievement over 8 years among children who met modified criteria for attention-deficit/hyperactivity disorder at 4–6 years of age. Journal of Abnormal Child Psychology.

[CR26] McArthur G (2022). Poor reading and anxiety (PRAX): Building a theory and practice. Australian Journal of Learning Difficulties.

[CR27] McArthur G, Badcock NA, Castles A, Robidoux S (2021). Tracking the relations between children’s reading and emotional health across time: Evidence from four large longitudinal studies. Reading Research Quarterly.

[CR28] Moll K, Landerl K (2009). Double dissociation between reading and spelling deficits. Scientific Studies of Reading.

[CR29] Moll K, Kunze S, Neuhoff N, Bruder J, Schulte-Körne G (2014). Specific learning disorder: Prevalence and gender differences. PLoS ONE.

[CR30] Mouzaki A, Protopapas A, Sideridis G, Simos P (2007). Examining the psychometric characteristics of a spelling test for students at the second, third, fourth and fifth grade of primary school. Epistimes Tis Agogis.

[CR31] Novita S (2016). Secondary symptoms of dyslexia: A comparison of self-esteem and anxiety profiles of children with and without dyslexia. European Journal of Special Needs Education.

[CR32] Novita S, Uyun Q, Witruk E, Siregar JR (2019). Children with dyslexia in different cultures: Investigation of anxiety and coping strategies of children with dyslexia in Indonesia and Germany. Annals of Dyslexia.

[CR33] Padeliadu, S., Antoniou, F., & Sideridis, G. (2019). *ΔΑΔΑ-Δοκιμασία Αξιολόγησης Δεξιοτήτων Ανάγνωσης* [DADA - Test for the assessment of reading skills]*.* Nicosia: Rocketlexia.

[CR34] Parhiala P, Torppa M, Eklund KK, Aro T, Poikkeus A, Heikkilä R, Ahonen T (2015). Psychosocial functioning of children with and without dyslexia: A follow-up study from ages four to nine. Dyslexia.

[CR35] Poole KL, Degnan KA, Harrewijn A, Almas AN, Fox NA, Henderson HA (2022). Trajectories of socially anxious behavior from age 5 to 13: Temperamental and sociocognitive pathways. Child Development.

[CR36] Raven, J. C. (1956). *Coloured progressive matrices, sets A, AB, B*. Lewis & Co., Ltd.

[CR37] Sideridis G, Antoniou F, Mouzaki A, Simos PG (2015). Raven’s coloured progressive matrices and vocabulary [in Greek].

[CR38] Slot, E. M., Van Viersen, S., De Bree, E., & Kroesbergen, E. H. (2016). Shared and unique risk factors underlying mathematical disability and reading and spelling disability. *Frontiers in Psychology*, *7*. 10.3389/fpsyg.2016.00803 10.3389/fpsyg.2016.00803 PMC4901067 27375508

[CR39] Swanson, J. M., Schuck, S., Porter, M. M., Carlson, C. L., Hartman, C. A., Sergeant, J. A., Clevenger, W., Wasdell, M. B., McCleary, R., Lakes, K. D., & Wigal, T. (2012). Categorical and dimensional definitions and evaluations of symptoms of ADHD: History of the SNAP and the SWAN Rating Scales. *The International Journal of Educational and Psychological Assessment*, *10*(1), 51–70. Retrieved January 17, 2023, from https://pubmed.ncbi.nlm.nih.gov/26504617/PMC4618695 26504617

[CR40] Tafa, E. (1995). *Tεστ ανίχνευσης της αναγνωστικής ικανότητας* [*Test for diagnosing reading ability*]. Ellinika Grammata.

[CR41] Tannock R, Brown TE (2008). ADHD with anxiety disorders. ADHD comorbidities: Handbook for ADHD complications in children and adults.

[CR42] Tarrasch R, Berman Z, Friedmann N (2016). Mindful reading: Mindfulness meditation helps keep readers with dyslexia and ADHD on the lexical track. Frontiers in Psychology.

[CR43] Westenberg PM, Drewes MJ, Goedhart AW, Siebelink BM, Treffers PDA (2004). A developmental analysis of self-reported fears in late childhood through mid-adolescence: Social-evaluative fears on the rise?. Journal of Child Psychology and Psychiatry.

[CR44] Willcutt EG, Nigg JT, Pennington BF, Solanto MV, Rohde LA, Tannock R, Loo SK, Carlson CL, McBurnett K, Lahey BB (2012). Validity of DSM-IV attention deficit/hyperactivity disorder symptom dimensions and subtypes. Journal of Abnormal Psychology.

[CR45] Zuppardo, L., Serrano, F., Pirrone, C., & Fuentes, A. R. (2021). More than words: Anxiety, self-esteem and behavioral problems in children and adolescents with dyslexia. *Learning Disability Quarterly*, 073194872110411. 10.1177/07319487211041103

